# Can Glucose Alarm Fatigue Threaten the Absolute Clinical Benefit of Continuous Glucose Monitoring in Optimal Glucose Management in Children and Adolescents with Type 1 Diabetes? A Narrative Review

**DOI:** 10.3390/children12121668

**Published:** 2025-12-08

**Authors:** Styliani Giza, Eleni P. Kotanidou, Vasiliki Rengina Tsinopoulou, Thekla Poukoulidou, Stergianna Ntouma, Assimina Galli-Tsinopoulou

**Affiliations:** Unit of Pediatric Endocrinology, Diabetes and Metabolism–Collaborative Sweet Center, 2nd Department of Paediatrics, School of Medicine, Faculty of Health Sciences, Aristotle University of Thessaloniki, AHEPA University General Hospital, Stilponos Kyriakidi 1, 54636 Thessaloniki, Greece; stellagiza@yahoo.com (S.G.); epkotanidou@auth.gr (E.P.K.); vasotsino@auth.gr (V.R.T.); tpoukoulidou@hotmail.com (T.P.); liandouma@yahoo.gr (S.N.)

**Keywords:** alarm fatigue, continuous glucose monitoring, insulin pump, type 1 diabetes, children, adolescents

## Abstract

**Highlights:**

**What are the main findings?**

- Alarm fatigue caused by increased use of diabetes technology is the most common cause of discontinuation of its use.
- It is a significant cause of compromised quality of sleep among children/adolescents and their parents/caregivers.

**What is the implication of the main finding?**

- There is a need for individualized education on alarms and psychological support.
- More accurate and user-friendly settings and algorithms will increase users’ trust in diabetes alarm systems.

**Abstract:**

Technology has brought about a revolution in the management of type 1 diabetes (T1D). The adoption of continuous glucose monitoring (CGM) and insulin pump therapy in the everyday life of children and adolescents with T1D is a real innovation and the most promising choice for optimizing glycemic control in this population. The incorporation of an alarm system, including notifications, alerts and alarms and warning patients and their parents about glucose levels and upcoming events interfering with safety, is an invaluable additional tool for better targeting euglycemia. However, in parallel with the clinical benefits of alarm systems in ameliorating metabolic control parameters, alarm fatigue was recorded as a phenomenon, negatively affecting the everyday lives of patients and their caregivers, and as a cause for rejecting or abandoning CGM or pump therapy treatment. There are a few data concerning the frequency, consequences and methods of eliminating alarm fatigue among children. As a result, we have conducted a narrative review to briefly present the basic philosophy of the existing CGM alarm systems and their positive effect on glycemic management, and focus on alarm fatigue; definition, frequency, effect on quality of life and sleep, not only of T1D pediatric patients but also of their families, and methods of elimination. Efforts to achieve a more reliable and accurate alarm system and educate on adapting personalized limits and positively interpreting them may protect the T1D pediatric population from alarm fatigue and prevent rejection or incomplete use of CGM and insulin pump as the therapeutic choice, ensuring the best glycemic control.

## 1. Introduction

Technology has brought about a revolution in the management of type 1 diabetes (T1D). The adoption of continuous glucose monitoring (CGM) and insulin pump therapy in the everyday life of children and adolescents with T1D is a real innovation and the most promising choice for optimizing glycemic control in this population [1,2].

The mission of CGM is to provide a measurement of interstitial glucose levels. It is offered not only as a single value but also as a short-term trend, long-term tracking, and daily pattern. In other words, it serves as a real-time (RT) glucose level detector, including not only the most commonly used RT-CGM systems (Dexcom San Diego, CA, USA, Metdronic Northridge, CA, USA) but also the flash CGM system, FreeStyle Libre 2 (Abbott Diabetes Care). Last one also functions as an RT-CGM system after the recent upgrade of its smartphone application. The only difference remains the prerequisite of scanning the sensor in order to obtain data [1,3]. It becomes evident that the knowledge of glucose levels at every single moment facilitates clinical decisions and makes them more targeted and optimal in avoiding hypoglycemia and hyperglycemia, and achieving good metabolic control. A lot of clinical [4,5] and real-world studies [6], as well as systematic reviews and meta-analyses [7,8], have clearly proven the beneficial effect of CGM on glycemic control optimization. Targeting further improvement in the context of glycemia and safety, CGM systems incorporated RT alarms, alerts, and notifications with the marketing of Guardian (Medtronic, Minneapolis, MN, USA) and STS™ (Dexcom) in the mid-2000s [9].

In parallel, the evolution of the insulin pump came to enhance the effect of CGM on metabolic control, with the hybrid closed loop (HCL) being the most promising entry in diabetes technology, implementing the communication of CGM with the insulin pump in clinical practice. There is an increasing number of studies underlining the increase in time in range (TIR) without a concomitant increase in time below range (TBR) among users [10,11,12,13,14]. Insulin pump as a stand-alone device is designed to have safety notifications, but, as a sensor-augmented or HCL system, its functionality was upgraded with certain alarms, intending to warn patients about detected changes in glucose levels or events expected to negatively affect their health status [15].

Although the whole alarm system keeps a leading role in glucose management, alarm fatigue has been increasingly and inevitably recorded among diabetes technology users. Alarm fatigue is defined as the situation of inappropriate responsiveness to warnings or complete deactivation of them after a period of frequent exposure to false or unnecessary alarms. Such a phenomenon declines trust in the CGM or insulin pump and puts at risk the subsequent utilization of the device. It is an expression of burnout, as receiving a great number of notifications, of which some are proven to be false, may be stressful and exhausting [16]. An alert is really meaningful if the user is educated on the significance and utilization of the information provided and responds properly. Otherwise, lack of knowledge predisposes the individual to alarm fatigue.

In this narrative review of the literature published since the early 2000s, we aimed to briefly present the basic philosophy of the existing CGM alarm systems and their positive effect on glycemic management and focus on alarm fatigue: definition, frequency, effect on quality of life (QoL) and sleep, not only of T1D pediatric patients but also of their families, and methods of elimination. We will focus not only on CGM and insulin pumps used independently, but also as part of HCL systems. There is a narrative review [17] on the benefits of a diabetes alarm system, but only scarce and scattered data on alarm fatigue, negatively affecting glycemic control, QoL and sleep of T1D pediatric patients and their parents.

### 1.1. Types of Alarms

An alarm system can concern CGM, an insulin pump or an HCL system and can be categorized in the following four groups: (1) CGM threshold that sends a notification when glucose levels rise above or fall below a value set by the user, necessitating an action, CGM predictive, based on specific algorithms, activated before hypoglycemia or hyperglycemia occurs and giving time for preventive measures and CGM velocity rate, released in case of increased velocity of glucose level change, (2) CGM maintenance to ensure proper system operation (calibration, lost sensor, expired sensor, change sensor, sensor error, weak signal, low transmitter battery and transmitter failure), (3) HCL system-specific (auto mode exit, auto mode sensor integrity failure, basal delivery resumption, maximum suspension, blood glucose required, open loop and calibration required for closed loop), and (4) insulin pump-specific (active temporary basal rate, suspension, low battery, battery out and low reservoir). Similarly, to the HCL system, in the case of the paired use of a connected insulin pen and CGM, there are notifications for missed or correction doses [9,16,18].

It is useful to understand the differences between alarms, informing oneself about an immediate issue that needs an urgent action related to medication or diet, and an alert warning about an upcoming risk situation where a safe action must be taken. Users are notified by sound or vibration with a varying repetition interval and an option for delay [3]. Reliability, validity, timeliness and perceptibility are the prerequisites for an alarm system to be successful [15].

### 1.2. Benefits of Diabetes Alarm System in Optimal Glucose Management

The mission of the diabetes alarm system is to warn one about out-of-range glucose levels and upcoming hypoglycemia or hyperglycemia, or notify users about technical issues. Informing users about glycemic events before or when they happen gives them the opportunity to understand their glucose level daily pattern and take action in order to attain euglycemia. Alarms serve as an additional valuable tool for optimizing glycemic management, reducing the incidence and severity of acute hypoglycemia and hyperglycemia, particularly among users who are not up to date on their glucose profile [16,18].

Besides protecting against hypoglycemia, it attains glycemic control, while hyperglycemia alerts have proven to reduce time above range (TAR) and decrease average glucose. The combined result is a decrease in glycemic variability and an increase in TIR [19,20,21,22]. Use of these alerts to adjust insulin delivery has the potential to improve glycated hemoglobin (HbA1c) [22].

Indeed, only the adaptation of a predictive hypoglycemia alert provided additional benefits over traditional low-threshold alerts, as TBR < 54 mg/dL, <70 mg/dL and TAR > 250 mg/dL decreased significantly regardless of the low-threshold alert setting [23]. Furthermore, in a prospective, observational study involving 47 children and adolescents with T1D, the possibility of optional alarms transitioning from FreeStyle Libre to FreeStyle Libre 2 for 14 days led to an increase in TIR and reductions in TAR, number of weekly hypoglycemic events and coefficient of variation [24].

The introduction of glucose alarms adds to the optimal glucose management and offers a sense of reassurance and safety [21,25,26]. Parents and young people report protection against hypoglycemic episodes as the primary cause of such feelings [25], while caregivers of children and adolescents using CGM find alarms useful in understanding the trending direction of glucose levels [27]. In the school environment, caregivers, teachers or school nurses consider an alarm system as a tool that simplifies glycemic management [22]. Accuracy improvement and the optional alarm feature of new-generation CGM systems can be rather useful for the adoption of this technology in the daily life of the pediatric population with T1D [28]. With the development of the HCL system, alarms ensure patients can take the minimal action necessary to maintain the optimal operation of the system, and manually control their glucose level in the presence of technical issues [9]. Furthermore, they are particularly useful for overnight management, as interruptions from alarms are fewer due to fewer glycemic events [29].

### 1.3. Disadvantages of Diabetes Alarm System

One of the main problems is alarm fatigue, which occurs due to too many and maybe false notifications that reduce the responsiveness of users to them. Another challenge is the embarrassment caused by notifications at an inappropriate time that produce a sense of loss of privacy [3]. Furthermore, children and adolescents, as well as their parents, were found to become upset with alerts, overtreating hypoglycemia with carbohydrates, even in the case of sensor-augmented insulin pumps. Such a reaction caused post-hypo peaks and threatened optimal glycemic control [30].

## 2. Materials and Methods

A comprehensive search was conducted on the PubMed/Medline database to identify relevant studies on the phenomenon of alarm fatigue in T1D children and adolescents and their parents and caregivers using one or more diabetes technology devices: CGM, insulin pump or HCL system. The literature search referred to manuscripts published between 1 January 2000 and 30 September 2025 using the following terms: “alarm fatigue”, “alarm frustration”, “alarm distress”, “device fatigue”, “technology fatigue”, “alert fatigue”, “notification fatigue”, “diabetes”, “glucose”, “CGM”, “insulin pump” and “HCL”. Exclusion criteria were articles with a non-English publication language, type 2 diabetes, exclusively adult population, clinical case reports, case series, editorials and letters to the editors. Studies including children and adolescents or parents/caregivers, observational and qualitative studies were included. The titles and abstracts of the retrieved manuscripts were scanned for relevance. Full-text articles of all the relevant studies were retrieved and reviewed. Comprehensive, narrative and systematic reviews were not included but used for the critical appraisal of studies included.

## 3. Results

**Figure 1:**
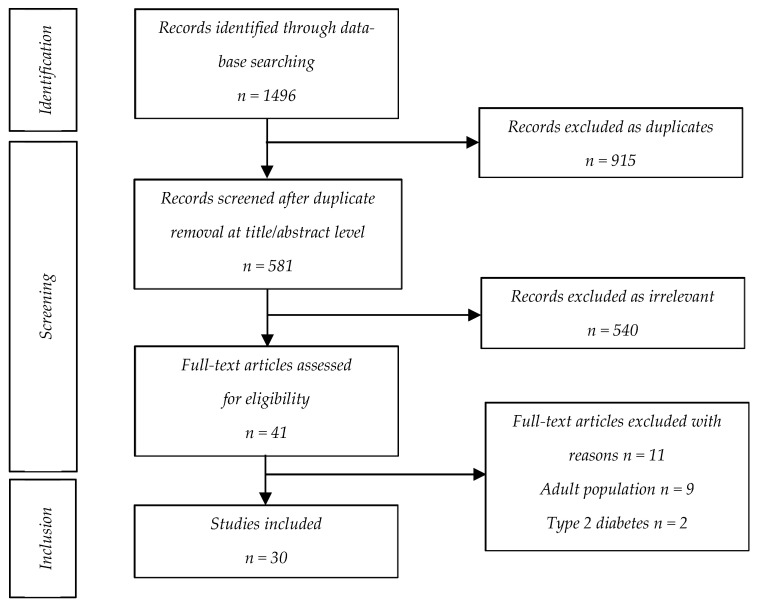
Flowchart of the literature review procedure (identification, screening and inclusion).

The initial literature search identified 1496 records, of which 915 were excluded as duplicates. Among the 581 reports that were retrieved, 540 were excluded as irrelevant, and 11 were excluded for reasons presented in detail in the screening flowchart. In the end, 30 articles were included in this narrative review (Figure 1). Table 1 presents the basic characteristics of the included studies and parameters recorded.

Studies recorded the impact of alarm fatigue among patients [18,29,31,33,34,36,43,44,45,47,49,53,55], their parents [22,27,32,35,38,42,46,50] or both [25,37,39,40,41,48,51,52,54]. Most of them were based either on an interview [25,29,31,35,38,42,46,51,52] or a questionnaire [27,32,33,34,37,39,41,43,54,55] and a few on CGM data [18,36,44,45,47,53]. The main studied parameters were quality of sleep [18,25,27,29,31,32,33,34,35,36,37,38,40,41,42,44,48,49,50,51] and QoL [22,25,29,31,33,34,37,39,41,43,45,46,49,51,52]. There were two studies being conducted with parental sleep [48] and QoL [42]. Frequency of alarm fatigue is either not recorded [22,25,29,31,32,33,34,35,36,37,38,50,51,52,53,54] or estimated in a rather variable way. Some studies aimed to detect alarm fatigue directly [18,27,40,41,42,46,48], while others used diabetes technology discontinuation percentage as an indirect estimation tool [39,43,47,49,55]. The frequency varied significantly from zero in a few studies that have not detected alarm fatigue at all [4,42,46] to 26–50% [18,39,41], while discontinuation percentage varied from 12 to 62% [39,43,45,49,55]. In the only study [48] that estimated parental sleep, alarm fatigue frequency was rather high (60%). However, not only the studies that have not detected alarm fatigue [4,42,46] but also those that recorded this phenomenon have identified the benefits of diabetes technology and more of CGM on glycemic control [22,25,29,31,34,47,52], hypoglycemia prevention and management [31,32,33,39,40,41,46,47,48,49,50,51,52,53,54], thus reducing the fear of hypoglycemia [39,40,41,46]. Although some studies detected the phenomenon of alarm fatigue, they underlined a positive effect on general well-being [29,31,32,33,34,37,46] and sleep [27,29,34,35,38,42,46].

The main reasons responsible for alarm fatigue concerned either everyday life [22,25,29,31,33,34,37,39,41,43,45,46,49,51,52] or sleep [18,25,27,29,31,32,33,34,35,36,37,38,40,41,42,44,48,49,50,51] and were provoked mainly by too many false alarms [22,47,49,54], although qualitative studies based on interviews also revealed factors that affected patients’ social well-being. Table 2 presents the main causes of alarm fatigue.

## 4. Discussion

### 4.1. Definition

As it was previously mentioned, alarm fatigue is the situation of reduced or absent responsiveness due to frequent false or unnecessary alarms, declining trust and long-term utilization of diabetes devices [16]. The concept of alarm fatigue can also include alarm embarrassment, an issue particularly affecting adolescents. It describes the negative feeling of loss of privacy experienced when users become the center of attention due to many alarms, and may also compromise further wearing a diabetes device. It is a kind of stigma produced when alerts go off in schools or social events [3]. Furthermore, too many alarms can produce disappointment, especially for users with sub-optimal self-management behaviors and/or a high HbA1c. They remind these patients of their diabetes diagnosis and imply a personal failure to achieve optimal blood glucose control [25]. In other words, there is a cycle of frustration, disappointment and embarrassment, leading to reduced adherence and maybe to discontinuation [26]. The patients discontinuing CGM as a result of alarm fatigue cannot take advantage of their beneficial effect on glycemic control [56,57].

### 4.2. Predisposing Factors

The high frequency of alarms increases the potential of fatigue [9,16]. It is not known how CGM alarm settings are associated with the number of alarms, and whether the alarm frequency alters patients’ responses and, as a result, is associated with alarm fatigue [21]. The quantity of alarms may depend on a number of factors, including how often glucose is desirable to be checked, the glucose variability, the awareness of hypoglycemia and the level of low threshold alarm, considering that the higher the alarm is set, the more alarms occur [16,21].

The average number of CGM alarms notifying pediatric users and their parents or caregivers is not known. Interdependently of it, a CGM user may perform from 15 to 48 glucose level checks a day [58], with the upper limit being approached in younger children due to the frequent and great fluctuations in blood glucose and the difficulty in recognizing or reporting symptoms of hypo- or hyperglycaemia [59,60].

A frequent cause of nuisance regarding hypoglycemia alarms, further predisposing users to alarm fatigue, is compression artifacts, caused by a decrease in glucose concentration in the interstitial fluid near the sensor tip when the CGM user sleeps on the CGM sensor [36].

It is not only the high frequency but also the sub-optimal accuracy of alerts that predispose to alarm fatigue and cause reduced adherence. Many alarms sound when glucose is already out of range, and some of them may be false or unnecessary [9,16]. Users feel overwhelmed and fatigued with the constant interruptions and tend to become unresponsive. As a result, alarms lose their urgent character [16,18].

It seems that alarm fatigue is caused by an imbalance between the sensitivity and specificity of CGM systems, as there is an inverse relationship between sensitivity and specificity [61]. Nowadays, some clinicians choose to counsel CGM users to set extremely low or high values as hypoglycemia and hyperglycemia alarm thresholds, respectively, in order to minimize early “nuisance alarms” [62,63]. On the other hand, there are clinicians who educate patients to choose a rather high hypoglycemia threshold as useful to detect and predict more hypoglycemic events [64,65]. The first approach favors specificity at the expense of sensitivity, while the second one promotes alarm fatigue by increasing sensitivity, allowing for more alarms that may be false. Such phenomena question the accuracy and credibility of CGM and make patients less reactive to alarms and less willing to retain them activated or even continue to use CGM [43,55]. In the same context, a too low threshold for hyperglycemia, intending to improve glycemic control, increases the risk of alarm fatigue [15]. However, retrospective studies of TITR do not support concerns that its use in clinical practice may contribute to alarm fatigue [66]. Scheinker et al. [53] found that hypothetical TITR alarms would trigger between 22% and 41% more frequently than TIR alarms. However, the use of robust TITR alarms, set to trigger on the basis of three consecutive readings below the threshold followed by three consecutive readings above the threshold, mitigated the increase in alarm frequency.

Decoding CGM alarms is not a black-or-white choice. The discrepancy between a low threshold alarm intended to prevent hypoglycaemia, more so when glucose is descending, and those requiring treatment, produces inconvenience and may be a cause of alarm fatigue, as there is a need for self monitoring blood glucose (SMBG) tests to confirm them [22].

### 4.3. Frequency

Alarm fatigue is rather frequent. In an anonymous survey among parents and caregivers of children with T1D using CGM, approximately 25% admitted that they remained unresponsive when the CGM alarms went off repeatedly, with potential poor outcomes for their children [59]. Among 85 children and adolescents between 5 and 18 years old with T1D, alarm fatigue was found to be the main reason for discontinuing CGM [43], or at least one of the reasons [32,67]. Even adolescent HCL users reported frustration around the number of alarms and notifications associated with the system [52]. “Alarms” was reported by 40% of pediatric HCL users as the third most common issue and recorded as a cause of HCL discontinuation [49]. If patients feel that the increased mental burden of responding to frequent notifications and alarms is too much, it could be a barrier to the adoption of this new technology [37,68]. In a study, among CGM ex-users, 50% reported too many alarms as the cause of CGM discontinuation. It is a matter of trust in the device to reassure continuing utilization [43]. Some patients reported anxiety in response to the notifications and occasionally disabled the hyperglycemia alarms to avoid discomfort from constant alerts [69].

### 4.4. Quality of Life

Some T1D patients believe that alarms may disrupt their daily life. About 40% of parents and caregivers of children with T1D using CGM reported the CGM device as a source of nervousness for them [59]. Interference in daily life was recorded among 38% of pediatric CGM users [39]. Alarms being released at inconvenient times draw attention to young users, producing embarrassment [51].

There are also many negative comments about alarms being annoying, as well as a life “living by alarms” [27]. Furthermore, they may promote a perception that CGM users lead a life dominated and dictated by their diabetes, with the alarms acting as reminders of their continuing struggle to cope with diabetes and achieve optimal glycemic control [25]. Alarms may produce unwelcome distractions at school, with many children reportedly switching alarms off in school due to concerns about drawing attention to themselves and distracting peers [22].

However, alarm fatigue is not a one-way potential for CGM users. As the integration of alarms into CGM systems improves, metabolic control can be achieved in the short term, without worsening the duration and quality of sleep, measured by actigraphy, not only in children and adolescents but also in their parents [24].

### 4.5. Quality of Sleep

Alarm fatigue can be expressed as nighttime sleep disruption not only for T1D children and adolescents but also for their families [24,25,41,70]. The transition from the SMBG to CGM did not protect T1D children and adolescents and their families from waking up multiple times during the night due to their CGM alarms. It is not only the fear of hypoglycemia but also caution about correction boluses overnight that disturbs sleep [59].

However, nocturnal hypoglycemia deserves special attention due to the urgency of coping with it, as it is reasonable for diabetes technology device users and their parents to be less willing to respond to alarms during the night and, as a result, more vulnerable to alarm fatigue. Parents and caregivers can be awakened many times during the night to administer insulin or provide carbohydrates for repetitive high or low glucose levels, respectively. Utilizing the 0–3 scale for the seven subscales of the Pittsburgh Sleep Quality Index, with 0 being the best and 3 being the worst, over 50% of parents and caregivers of children with T1D using CGM scored a 2 or 3 on the sleep disturbance subscale. About 70% of them scaled their sleep quality as fairly bad or very bad. It is interesting that 30% of caregivers worried they would incorrectly set alarm limits on the CGM device at night [59]. In a qualitative study, caregivers of minors with T1D reported waking frequently to check CGM, while several ones confessed that they additionally set clock alarms to make sure that their child’s glucose was in range, despite having a CGM system, revealing a deficiency of trust in CGM alarms, not fulfilling their mission [71].

In another study, disrupted sleep was commonly reported, with 73% of parents/caregivers reporting waking up because of diabetes technology. Of these, 54% reported waking at least four times a week, and one of the main reasons reported was CGM alarms (38%). About 10% reported false alarms occurring more than once a week. However, participants graded the impact of diabetes technology for their child as generally positive [60].

Overnight awakening due to CGM alerts and notifications was also reported in two other studies, performing qualitative narrative analysis of CGM and evaluating its impact on parental sleep [27,35].

A great number of false alarms were detected [62]. Awaking was more common among parents “sleeping lightly” and especially mothers [48]. The high frequency of alarms is a cause of nocturnal awakenings, poor sleep quality, and reduced long-term device use [18]. Overnight alarm fatigue preserves the vicious cycle of unresponsiveness as patients with hypoglycaemia react only to 29% of alarms [27], while parents only wake to 37% of them [36].

Even insulin pump and HCL system users may suffer from alarm fatigue. Even with closed-loop systems, T1D patients experience nocturnal hypoglycemia 25% of the time [41]. Waking up due to alarms was reported as frustrating for sensor-augmented pump therapy users, because it was frequently unclear why they went off [51]. In regard to HCL, the causes of nocturnal alarms were evenly distributed among the four different types of alarms. During the first 2 weeks after initiation of HCL mode, the mean number of nocturnal alarms increased significantly, followed by a steady decline in HCL mode and sensor use. Nocturnal alarms may be a contributing factor to the decline in system use, as has been described previously [18].

### 4.6. Management

#### 4.6.1. General Principles

In order to successfully face the phenomenon of alarm fatigue, alarms should be designed in a way that they fulfill their mission, to reliably detect and warn for upcoming events that require action [72], in the most user-friendly way. In other words, increasing accuracy and reliability translates to less alarm fatigue and greater safety and more trust in the device. Their adjustment must achieve a balance between safety and QoL, avoiding unnecessary notifications that can generate alarm fatigue [16].

#### 4.6.2. Alarm Deactivation

The option to turn off alarms has the potential to reduce alarm fatigue among users who are more prone to experience annoyance at the expense of treatment satisfaction and adherence [3]. The flexibility to turn off the alarms when needed and eliminate disruptions at inappropriate times were recorded as the two greatest benefits of optional alarms, offering a sense of control, freedom and strength [26]. Some CGM users deactivate all alarms overnight and swithch on only hypoglycemia alerts as a solution to cope with alarm fatigue [69].

#### 4.6.3. Development of Algorithms

The development of algorithms may serve in this context [16], for learning individual glucose profiles and providing earlier and more accurate prediction of glucose levels, focused on hypoglycemia [33,73,74]. Maximizing model performance for glucose risk prediction and management is crucial for reducing the burden of alarm fatigue on CGM users [74]. There is a need to develop more accurate and reliable CGM systems. Even the compression artifacts should be eliminated [60].

#### 4.6.4. Selection of Settings

Furthermore, there is a need for efforts to make some alarms and alerts more discreet, like a mobile text notification, in order to eliminate social and emotional embarrassment. In this context, vibrating alarms possess an advantage compared with audible alarms, as they are noticeable even in a noisy environment and they cause less embarrassment. Different kinds of alarms should be distinctive for easier and quicker recognition of the nature and severity of the problem, with a gradual escalation in case the users remain unresponsive in order to increase the possibility of reaction [16]. However, during the night, it is important for the children or adolescents and their parents/caregivers to be aware of the alarms, as many of them are not heard at night by those who oversee their treatment [27,75]. Sharing alarms with parents may reduce some of the load of responsiveness undertaken by the users, and systems have been developed to support that need [75]. Furthermore, the selection of rather necessary hypo-and hyperglycemia threshold alarms over velocity rate alerts may be an attempt to prevent alert fatigue or to limit the number of alert disturbances both in the classroom and overnight [22].

#### 4.6.5. Threshold Levels

It is important for alarms to overcome the role of automated reminders and be connected with real-life circumstances, promoting targeted and supportive interventions. CGM is something more than a “hypoglycemia or hyperglycemia detector”. It is a monitoring device that has the ability not only to detect but also to avoid hypoglycemia and hyperglycemia. There are periods of time when an “alarm not necessitating treatment” may be valuable, such as in severe hypoglycemia unawareness [22]. On the other hand, in periods of fatigue, customization of alarms to provide meaningful support in the form of notifications is recommended. Health professionals should help patients and their families handle any difficulty associated with alarm fatigue in order to overcome any resistance to use and take advantage of alarms, or avoid their deactivation or diabetes technology rejection [3]. At last, it is a matter of choice between risk and nuisance when the scale moves towards timeliness of excursion detection or false alarm, respectively [61].

Therefore, it is essential to individualize the activation threshold for each person, ensuring a balance between effectiveness and OoL. Patient-centered alarm configuration in daily life includes even varying alarm settings according to time of day or special situations such as physical activity or sick days [16] and adjusting them according to the course of glycemic control [22]. At first, it is recommended to set extremely low and high hypoglycemia and hyperglycemia alarm thresholds, respectively, to eliminate false alarms in favor of effective long-term use. Setting higher hypoglycemic thresholds predisposes users to the alarm fatigue phenomenon, not necessarily improving glycemic control, as alarms are ignored and no action is undertaken. The option to delay the hyperglycemia alert has been shown in some studies to reduce the incidence of hypoglycemia due to overcorrection by avoiding unnecessary insulin administration [75]. As glucose control improves with concomitant reduction in TBR, a more aggressive level of 60 and 140 mg/dL for hypo- and hyperglycemia threshold could be selected, respectively. On the other hand, in a patient without optimal glycemic control, such alarm settings would be out of scope and rather bothersome [22]. Furthermore, the discrimination of hypo-and hyperglycemia in regard to the urgency of treatment is in favor of a delay in the alarm release in the second situation.

An alarm threshold of 75 mg/dL was the optimal cutoff for hypoglycemia alarms in order to achieve a TBR < 1% as it offered the essential time for CGM users to react and overcame the obstacle of the deficits in the counter-regulatory mechanisms of euglycemia restoration, and kept the false positive rate at a low level. In the majority of cases, a hypoglycemic alarm threshold at around 70 mg/dL reduces TBR > 50% [21]. The alarm threshold should be adjusted according to age, medical history, and hypoglycemia awareness, as well as the frequency of hypoglycemic events during certain periods [75].

Hyperglycemia alerts are rather helpful in young children with a greater risk of diabetic ketoacidosis, for insulin pump users, and for patients with good glycemic control who want to further ameliorate glycemic parameters [75]. An alarm threshold of 170 mg/dL was the optimal cutoff for hyperglycemia alarms in order to achieve a TAR < 5% and HbA1c ≤ 7%. Gradually lowering hyperglycemia alarm thresholds leads to greater numbers of alarms, predisposing users to alarm fatigue. Thus, this practice could be considered to be adapted in the daily life of CGM users who need to further optimize HbA1c or reduce TAR, without increasing TBR, but until the level where there is no further improvement in glycemic control. Then, under the risk of alarm fatigue, alarm adjustments should stay stable or even loosen moderately [21].

#### 4.6.6. Personalized Approach

There is a need for substantial personalized training aiming to strengthen patient confidence, reduce disease-related anxiety and encourage active participation in daily self-care. Clear communication reduces anxiety, facilitates learning and promotes greater adherence to treatment. It is important to optimize alarm settings with respect to each user’s preferences regarding frequency, timing, and content of notifications [69], in order to improve user engagement and long-term adherence [76,77]. For HCL users, there is a need for re-education in the timing of sensor calibrations, reacting to system alerts and setting alerts to reduce the number of nuisance alarms [78]. Identifying the source of alarms, especially nocturnal ones, on an individual basis, is essential in order to make the appropriate clinical interventions that can alleviate the recurrence of specific alarms for each person [18]. It is essential to configure only the alerts necessary for decision-making and adjust them to each person. This can be mitigated by adjusting thresholds according to time, activating vibration mode, or temporarily disabling some alerts. Diabetes education is the key for the user to know how to act in response to each alert and avoid unnecessary corrections.

#### 4.6.7. Recommendations

There are specific recommendations regarding CGM glucose alarms for individuals who are new to CGM. The process of adaptation in daily life passes through education on alarms, psychological support to handle related symptoms and concerns, regular review of glucose data, a gradual approach to set up alarms starting with the most important ones and sharing data with parents/caregivers [17].

Directed towards T1D patients resistant to using CGM alarms, Miller et al. [3] published a five-step practical approach. After uncovering patients’ anxieties, health professionals should discuss how the upcoming change could meet their needs. It is important to analyze the benefits of CGM, how they can integrate it into their lives and use optional alarms/alerts. For those patients, the best way is to make them experience in their daily life how alarms have the ability to eliminate anxiety and improve QoL. This opportunity may be made available gradually, choosing to turn on and adjust alarms properly without pressure and deactivate them at any time. Patients should be encouraged to review their glucose reports and daily graphs in order to identify trends, when and why they occur, and how to prevent hypo- or hyperglycemic events. This is the critical point to offer the alternative of alarms, if they can understand the significance of potential warnings on upcoming glycemic events, to take action. In case the patients choose to use alarms, counseling on effective utilization according to their needs is suggested. By analyzing their glucose pattern, glucose thresholds are properly set, and alarms are appropriately activated. There may be differentiation from patient to patient according to the level of glycemic control, but also, in every single patient, throughout the day according to the current glucose pattern, the aimed glucose target and social circumstances. Finally, there may be an adaptation over time or to special cases.

## 5. Conclusions

In summary, with the utilization of technology for optimal glucose management in T1D, the phenomenon of alarm fatigue has arisen in parallel. It substitutes a situation of inappropriate response to a high frequency of alarms, even more so when some of them are proven to be false. Unfortunately, it is not limited only to pediatric patients, as it also affects their parents/caregivers. A cycle of frustration, disappointment and embarrassment is evoked and maintained, leading to reduced adherence and maybe to the discontinuation of diabetes technology devices. As alarms are a valuable tool of CGM, insulin pumps and HCL systems for optimal glycemic management, it is essential to find a way to reinforce their incorporation in the everyday life of children and adolescents with T1D. There is a need for an individualized approach, including education on alarms, psychological support, regular review of glucose data, gradual adoption of personalized alarms and sharing of the load of handling with parents/caregivers. This kind of approach should be a lifelong process. Furthermore, efforts should be made in the direction of improved accuracy and development of user-friendly settings and algorithms to further increase users’ trust in diabetes alarm systems. Only then, optimal and long-term utilization of alarms can ensure the best possible glycemic control for every patient without compromising QoL. Although alarm fatigue is a phenomenon that cannot be ignored, it is not able to threaten the undeniable positive effect on glycemic control.

## Figures and Tables

**Table 1:** Basic characteristics of the included studies and parameters recorded.

No	Author, Year	Study Type	Tool	Country	Population	Sample Size	Age (Years)	Type of Technology	Alarm Fatigue Concept	Alarm Fatigue Frequency	Collateral Effects of Alarm Fatigue	Benefits
1	Aljuhani, 2024 [31]	CrSC	Interview	Saudi Arabia	Patients	121	2–18	CGM	Sleep, QoL	NR	-	Better impact than SMBG on glycemic control, hypo- and hyperglycemic episodes and psychosocial factors
2	Barnard, 2016 [32]	CrSC	Questionnaire	USA	Parents	100	1–17	CGM, CSII, HCL	Sleep	NR	Disrupted sleep, waking	Reduced severity and frequency of hypoglycemia, easier to achieve blood glucose targets, generally positive impact for children
3	Barnard, 2017 [33]	CrSC	Questionnaire	UK, Germany, Austria	Patients	26	6–18	CGM, CSII, HCL	Sleep, QoL	NR	Disturbing alarms, particularly overnight	Hypoglycemia warning, improved general well-being, improved sleep
4	Beato-Víbora, 2020 [34]	PC	Questionnaire	Spain	Patients	22	13–18	HCL	Sleep, QoL	NR	9% reduction in poor sleep quality	Improved sleep and QoL and glycemic control, diabetes treatment satisfaction
5	Bispham, 2021 [35]	qualitative	Interview	USA	Caregivers	14	7–15	CGM, CSII	Sleep	NR	Overnight disturbance, poor sleep, sleep anxiety of children	Safety, more sleep
6	Buckingham, 2005 [36]	CrSC	CGM data	USA	Patients	20	4–17	CGM	Sleep	NR	Poor sleep quality, waking, willingness to use device	NR
7	Burckhardt, 2018 [37]	PC	Questionnaire	Australia	Patients, Parents	49 Patients, 49 Parents	2–12	CGM	Sleep, QoL	NR	-	Improved QoL and parental sleep
8	Burckhardt, 2019 [38]	CrSC	Interview	Australia	Parents	20	5–12	CGM, CSII	Sleep	NR	-	Reassurance of alarms, waking only momentarily, able to return to work, improved sleep, increased peace of mind during the night, increased freedom and confidence
9	Cemeroglu, 2010 [39]	RC	Questionnaire	USA	Patients, Parents	40	3–25	CGM, HCL	QoL	38–48%	Interference in daily routine by CGM alarm (38%), annoyance/irritation from the CGM alarm (48%), discontinuation after 1 month (12%)	Prevention of hypoglycemia, decrease in hypoglycemia-related anxiety
10	Cobry, 2020 [40]	PC	Questionnaire, actigraphy, sleep diary	USA	Patients, Parents	37 patients, 37 parents	10–17	HCL	Sleep	0	-	Significantly improved glucose monitoring satisfaction after 3 months of HCL use, moderate effect sizes for improvements in adolescents’ sleep efficiency and fear of hypoglycemia and in parents’ self-reported sleep quality
11	Cobry, 2024 [18]	PC	CGM data	USA	Patients	76	7–24	CGM, HCL	Sleep	35,3% HCL, 26% CGM	Nocturnal awakenings, poor sleep quality, reduced long-term device use	NR
12	Dubose, 2021 [41]	PC	Questionnaire	USA	Patients, Parents	80	<18	CGM, HCL	Sleep, QoL	>50%	Sleep interruption at night, increasingly bothersome during the day	Decreased fear of hypoglycemia
13	Elbalshy, 2020 [42]	CrSC	Interview	New Zealand	Parents	12	<16	CGM	Sleep, QoL of parents	0	-	Better parental QoL, better parental sleep quality/quantity, improved sleep quality for children and safety
14	Erie, 2018 [22]	CrSC	Survey	USA	Parents, Daytime caregivers	33 parents, 17 daytime caregivers	2–17	CGM	Alarm frequency, QoL	NR	High frequency	Mitigation of glycemic variation, HbA1C improvement, teachers felt that CGM use in schools was useful
15	Farfel, 2020 [43]	RC	Questionnaire	Israel	Patients	85	5–18	CGM, CSII	QoL	54%	CGM discontinuation	NR
16	Gandrud, 2004 [44]	Open label	CGM data	USA	Patients	45	7–17	CGM	Sleep	NR	Sleep disruption (32%), false positive for nocturnal hypoglycemia alarms (50%)	Helpful (74%)
17	Harvengt, 2025 [45]	RC	CGM data	Belgium	Patients	67	<18	CGM	QoL	NR	Low positive predictive value for hypoglycemia, increased false alarms	Reduced risk of alarm fatigue due to sufficient spacing of false alarms through time
18	Iturralde, 2017 [29]	PC	Interview	USA	Patients	17	14–18	CGM, HCL	Sleep, QoL	NR	Responding to alarms	Improved sleep, QoL, glycemic control
19	Karakuş, 2021 [46]	Qualitative	Interview	Turkey	Caregivers	15	<9	CGM	Sleep, QoL	0	-	Improved children’s daily life, safe and comfortable night, removal of hypoglycemia fear, better metabolic control
20	Lal, 2019 [47]	PC	CGM data	USA	Patients	26	<18	CGM, HCL	Alarm frequency	62%	At 1 year, 62%, discontinued Auto Mode for reasons due to sensor issues, including alarms	Improved glycemic control
21	Lawton, 2017 [25]	Qualitative	Interview	UK	Patients, Parents	5 Patients, 9 Parents	13–20	CGM, CSII	Sleep, QoL	NR	Poor or interrupted sleep and/or unwelcomed distractions at school, tangible and difficult reminder of diabetes and of their struggles to achieve optimal blood glucose control	Improved glycemic control, increased confidence
22	Macaulay, 2020 [48]	PC	Interview Actigraphy	New Zealand	Patients, Parents	16 patients, 20 parents	1–17	CGM, CSII	Sleep of parents	60%	Parental sleep disturbance, awakenings, perception of “sleeping lightly”	NR
23	Messer, 2020 [49]	PC	Questionnaire, interview	USA	Patients	92	8–25	CGM, HCL	Sleep, QoL	40%	HCL discontinuation	NR
24	Oser, 2017 [50]	Qualitative	Blog	USA	Parents	140 blogs, 663 comments	4–16	NR	Sleep	NR	Lost sleep	NR
25	Pickup, 2025 [27]	CrSC	Questionnaire	UK	Parents	50	3–17	CGM, CSII	Sleep	15%	Alarms being annoying, intrusive, disturbing, life “living by alarms”	Some slept more easily, with less disturbance and feeling of safety; some were unable to sleep properly if not using CGM.
26	Rashotte, 2014 [51]	Qualitative	Interview	Canada	Patients, Parents	7 Patients, 9 Parents	13–17	CGM, CSII	Sleep, QoL	NR	CGM alarming at inconvenient times, causing sudden unwanted visibility, sense of being different; CGM interfering with routines or interrupting “important times,” loss of flexibility, spontaneity when required recalibration or new sensor was needed.	NR
27	Roberts, 2022 [52]	CrSC	Interview	Australia	Patients, Parents	17 patients, 10 parents	12–25	HCL	QoL	NR	Frustration around number of alarms	Improvement in glucose levels and increased independence with diabetes management
28	Scheinker, 2025 [53]	RC	CGM data	USA	Patients	493	<18	CGM, HCL	Alarm frequency	NR	NR	NR
29	Tansey, 2011 [54]	PC	Questionnaire	USA	Patients, Parents	208 patients, 192 parents	8–18	CGM	Alarm frequency	NR	Decreased overall satisfaction	Ability to detect hypoglycaemia and self-correct out-of-range glucose levels
30	Wong, 2014 [55]	PC	Questionnaire	USA	Patients	457	<18	CGM	Alarm frequency	27%	CGM discontinuation	NR

CGM: continuous glucose monitoring, CrSC: cross-sectional cohort, CSII: continuous subcutaneous insulin infusion, HbA1C: glycated hemoglobin, HCL: hybrid closed loop, NR: not reported, PC: prospective cohort, QoL: quality of life, RC: retrospective cohort, SMBG: self monitoring blood glucose, UK: United Kingdom, USA: United States of America.

**Table 2:** Predisposing factors of alarm fatigue.

Predisposing Factors of Alarm Fatigue
◆ High frequency of false alarms
◆ Annoyance/irritation during everyday life
✔ Unwanted visibility
✔ Interruption of “important” moments
◆ Overnight disturbance, poor sleep quality
◆ Sense of being different
◆ A life living with alarms

## Data Availability

No new data were created or analyzed in this study.

## Abbreviations

The following abbreviations are used in this manuscript:

CGM	continuous glucose monitoring
CrSC	cross-sectional cohort
CSII	continuous subcutaneous insulin infusion
HbA1C	glycated hemoglobin
HCL	hybrid closed loop
NR	not reported
PC	prospective cohort
QoL	quality of life
RC	retrospective cohort
SMBG	self monitoring blood glucose
TAR	time above range
TBR	time below range
TIR	time in range
TITR	time in tight range
T1D	type 1 diabetes
UK	United Kingdom
USA	United States of America
